# Purification, Identification, Activity Evaluation, and Stability of Antioxidant Peptides from Alcalase Hydrolysate of Antarctic Krill (*Euphausia superba*) Proteins

**DOI:** 10.3390/md19060347

**Published:** 2021-06-17

**Authors:** Shuang-Yi Zhang, Guo-Xu Zhao, Shi-Kun Suo, Yu-Mei Wang, Chang-Feng Chi, Bin Wang

**Affiliations:** 1 Zhejiang Provincial Engineering Technology Research Center of Marine Biomedical Products, School of Food and Pharmacy, Zhejiang Ocean University, Zhoushan 316022, China; zsydoubleone@outlook.com (S.-Y.Z.); xuzhao1109@sina.com (G.-X.Z.); 13275896859@163.com (S.-K.S.); Wangyumei731@163.com (Y.-M.W.); 2 National and Provincial Joint Laboratory of Exploration and Utilization of Marine Aquatic Genetic Resources, National Engineering Research Center of Marine Facilities Aquaculture, School of Marine Science and Technology, Zhejiang Ocean University, Zhoushan 316022, China

**Keywords:** Antarctic krill (*Euphausia superba*), peptide, antioxidant activity, stability

## Abstract

For utilizing the largest source of marine proteins, Antarctic krill (*Euphausia superba*) proteins were defatted and hydrolyzed separately using pepsin, alcalase, papain, trypsin, and netrase, and alcalase hydrolysate (EPAH) showed the highest DPPH radical (DPPH·) and hydroxyl radical (HO·) scavenging activity among five hydrolysates. Using ultrafiltration and chromatography methods, fifteen antioxidant peptides were purified from EPAH and identified as Asn-Gln-Met (NQM), Trp-Phe-Pro-Met (WFPM), Gln-Asn-Pro-Thr (QNPT), Tyr-Met-Asn-Phe (YMNF), Ser-Gly-Pro-Ala (SGPA), Ser-Leu-Pro-Tyr (SLPY), Gln-Tyr-Pro-Pro-Met-Gln-Tyr (QYPPMQY), Glu-Tyr-Glu-Ala (EYEA), Asn-Trp-Asp-Asp-Met-Arg-Ile-Val-Ala-Val (NWDDMRIVAV), Trp-Asp-Asp-Met-Glu-Arg-Leu-Val-Met-Ile (WDDMERLVMI), Asn-Trp-Asp-Asp-Met-Glu-Pro-Ser-Phe (NWD-DMEPSF), Asn-Gly-Pro-Asp-Pro-Arg-Pro-Ser-Gln-Gln (NGPDPRPSQQ), Ala-Phe-Leu-Trp-Asn (AFLWA), Asn-Val-Pro-Asp-Met (NVPDM), and Thr-Phe-Pro-Ile-Tyr-Asp-Tyr-Pro-Gln (TFPIYDPQ), respectively, using a protein sequencer and ESI/MS. Among fifteen antioxidant peptides, SLPY, QYPPMQY and EYEA showed the highest scavenging activities on DPPH· (EC_50_ values of 1.18 ± 0.036, 1.547 ± 0.150, and 1.372 ± 0.274 mg/mL, respectively), HO· (EC_50_ values of 0.826 ± 0.027, 1.022 ± 0.058, and 0.946 ± 0.011 mg/mL, respectively), and superoxide anion radical (EC_50_ values of 0.789 ± 0.079, 0.913 ± 0.007, and 0.793 ± 0.056 mg/mL, respectively). Moreover, SLPY, QYPPMQY and EYEA showed strong reducing power, protective capability against H_2_O_2_-damaged plasmid DNA, and lipid peroxidation inhibition ability. Furthermore, SLPY, QYPPMQY, and EYEA had high stability under temperatures lower than 80 °C, pH values ranged from 6–8, and simulated GI digestion for 180 min. The results showed that fifteen antioxidant peptides from alcalase hydrolysate of Antarctic krill proteins, especially SLPY, QYPPMQY and EYEA, might serve as effective antioxidant agents applied in food and health products.

## 1. Introduction

Antarctic krill (*Euphausia superba*) is a crucial marine biological resource distributed in the Antarctic Ocean [1,2]. Its total biomass is up to 6.5–10 million tons and has been thought as the largest underexploited resource of the ocean [3,4]. Presently, Antarctic krill is thought as an abundant and high-quality resource for various food and health-care products because some bioactive substances, including oil, peptides and protein, astaxanthin, and chitin, have been prepared from Antarctic krill (*E. superba*) and its processing by-products [5,6,7,8,9,10].

Bioactive peptides are composed of 2 to 20 amino acid residues and released from their parent proteins using different methods of hydrolysis, including enzymatic hydrolysis, chemical hydrolysis, and biological fermentation [11,12,13]. Beyond the recognized nutritional value, bioactive peptides exhibited various biological functions, including hypolipidemic [14], hypotensive [15], anticoagulant [16], anticancer [17,18], and antimicrobial activities [19]. Among the most studied bioactive peptides, antioxidant peptides derived from marine living resources and their processing by-products, such as yellowfin tuna (*Thunnus albacares*) skin [20], monkfish muscle [21,22], red tilapia (*Oreochromis* sp.) scale [23], miiuy croaker swim bladder [24], Skipjack tuna bone and head [25,26], and mackerel muscle [13], exhibit excellent capacity for inhibiting lipid peroxidation and scavenging reactive oxide species (ROS). EDIVCW and YWDAW from the protein hydrolysate of monkfish muscle showed strong radical scavenging activity and equivalent capability on controlling lipid peroxidation with glutathione (GSH). Moreover, EDIVCW and YWDAW showed positive protective function on H_2_O_2_-damaged HepG2 through increasing the activity of antioxidant enzymes (superoxide dismutase and glutathione peroxidase) and decreasing the contents of ROS and malondialdehyde (MDA) [22]. Similarly, the antioxidant hexapeptide of FPYLRH from the swim bladder hydrolysate of miiuy croaker (*Miichthys miiuy*) could improve the viability of H_2_O_2_-damaged HUVECs through increasing the activity of intracellular antioxidant enzymes and lowering the levels of ROS, MDA, and nitric oxide [24]. ICRD and LCGEC from the roe protein hydrolysate of skipjack tuna (*Katsuwonus pelamis*) could decrease the apoptosis of HaCaT cells induced by ultraviolet-B treatment and altered Keap1/Nrf2-ARE pathway transcription [27]. EDYGA from soft-shelled turtles was confirmed as the most potent ARE-luciferase inducer because it could increase the Nrf2 level through down-regulating Keap1 [28]. Therefore, antioxidant peptides derived from marine resource have drawn worldwide attention due to its huge potential applied in drugs, health-care products, as well as for food industries of quality control [11,29].

As the most abundant marine biological resource, the utilization of Antarctic krill proteins has been continuously studied, and bioactive peptides from Antarctic krill proteins, including angiotensin converting enzyme (ACE) inhibitory peptides, high Fisher value oligopeptides, anti-osteoporotic peptides, metallic element chelating peptides, and dipeptidyl peptidase IV (DPP-IV) inhibitory peptides, have gathered considerable attention because of their significant bioactive activities [2,4,8,30,31]. Wang et al. (2019) prepared peptides through hydrolyzing Antarctic krill using neutral proteinase and the peptides could down-regulate the expression of hypoxia-inducible factor-2α and its downstream genes to ameliorate the cartilage degeneration of the medial meniscus mouse model [8]. Zhao et al. (2019) isolated eight ACE inhibiting peptides from the trypsin hydrolysate of Antarctic krill protein and the tripeptide of FAS could adjust the contents of nitric oxide and endothelin-1 of HUVEC and correct the endothelial cell dysfunction [32]. KVEPLP, PAL, and IPA from the hydrolysate of Antarctic krill protein using animal proteolytic enzymes could be used to manage hypertension and diabetes because of their strong DPP-IV and/or ACE inhibitory activity [6,33]. Xia et al. (2015) and Han et al. (2018) reported that treatment with phosphorylated peptides from Antarctic krill could significantly prevent the decrease in bone mass and improve porous bone structures and biochemical characteristics of ovariectomized Sprague Dawley rats [30,34]. Hou et al. (2018) and Sun et al. (2021) found that trypsin hydrolysate of Antarctic krill protein and VLGYIQIR could be applied as a novel calcium and zinc supplement [2,31]. Equally, antioxidant peptides from Antarctic krill protein are rarely reported. Then, this study was mainly to isolate and characterize antioxidant peptides from protein hydrolysates of Antarctic krill. Moreover, the antioxidant activity and stability of isolated antioxidant peptides were systematically investigated.

## 2. Results and Discussion

### 2.1. Preparation of Protein Hydrolysates of Antarctic Krill and Their Radical Scavenging Activities

**Figure 1:**
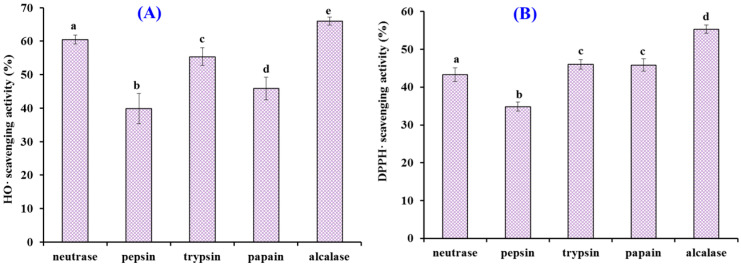
HO· (**A**) and DPPH· (**B**) scavenging activities of enzymatic hydrolysates from Antarctic krill (*E. superba*) proteins at the concentration of 5.0 mg/mL. All the results were triplicates of mean ± SD. ^a–e^ Columns with the same superscripts indicate no significant difference (*p* > 0.05).

The specificity of protease determines its hydrolysis site, thus significantly affecting the degree of hydrolysis and biological activity of protein hydrolysate [4,35,36]. Therefore, defatted Antarctic krill powder was separately hydrolyzed with alcalase, trypsin, neutrase, pepsin, and papain, and the radical scavenging activity of five protein hydrolysates is shown in Figure 1. At the concentration of 5.0 mg/mL, the HO· and DPPH· scavenging ratios of alcalase hydrolysate were 65.99 ± 1.22% and 55.32 ± 1.08%, respectively, which were significantly higher than those of trypsin, neutrase, pepsin, and papain hydrolysates (*p* < 0.05). Alcalase is an endo-protease of the serine type and can degrade most amido bonds of protein molecules, and is usually as a tool enzyme to prepare antioxidant peptides from marine protein resources, such as croceine croaker muscle [37], bluefin tuna heads [38], and swim bladders of miiuy croaker [39]. Therefore, the alcalase hydrolysate (named EPAH) of Antarctic krill proteins was selected for further study.

### 2.2. Preparation of Antioxidant Peptides from EPAH

**Figure 2:**
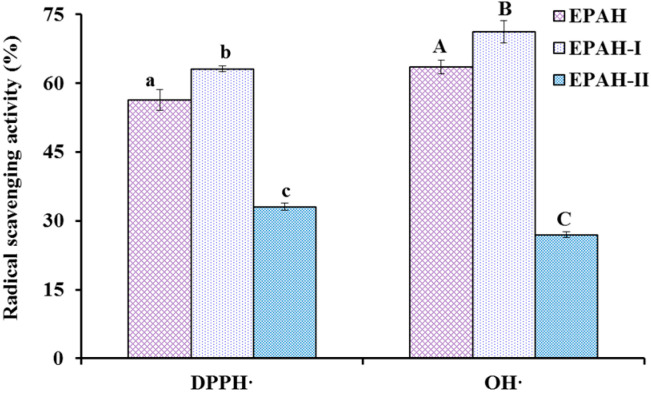
HO· and DPPH· scavenging activity (%) of alcalase hydrolysate and its two fractions from Antarctic krill (*E. superba*) proteins at the concentration of 5.0 mg/mL. All the results were triplicates of mean ± SD. ^a–c or A–C^ Columns with the same superscripts of this type indicate no significant difference (*p* > 0.05).

Using the MW cut-off membrane of 3.5 kDa, EPAH was divided into two peptide fractions including EPAH-I (MW < 3.5 kDa) and EPAH-II (MW > 3.5 kDa), and their antioxidant activity is presented in Figure 2. At the concentration of 5.0 mg/mL, HO· and DPPH· scavenging activities of EPAH-I were 71.17 ± 1.52% and 63.09 ± 2.40%, respectively, which were significantly higher than those of EPAH (65.99 ± 1.22% and 55.32 ± 1.08%) and EPAH-II (27.01 ± 0.59% and 33.05 ± 0.76%) (*p* < 0.05). MW is considered to be one of the main factors affecting the antioxidant activity [11,37]. Li et al. (2013) and Chi et al. (2016) reported that the antioxidant capacities, including radical scavenging activity and reducing power of collagen hydrolysates from cartilages, were negatively correlated with the logarithm of their average MWs [40,41]. Therefore, EPAH-I with smaller MW showing high radical scavenging activity was in line with these previous reports.

**Figure 3:**
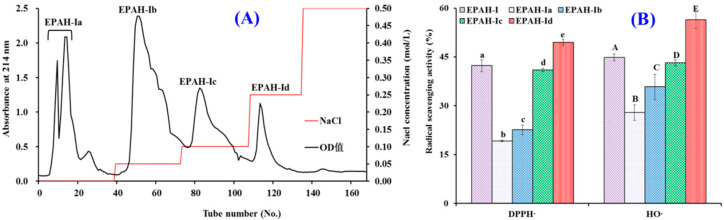
DEAE-52 cellulose chromatographic diagram (**A**) of EPAH-I and radical scavenging activity of EPAH-I and its four fractions (EPAH-Ia to EPAH-Id) at 2.0 mg/mL (**B**). All the results were triplicates of mean ± SD. ^a–e or A–E^ Columns with the same superscripts of this type indicate no significant difference (*p* > 0.05).

To obtain the sub-fractions with higher antioxidant activity, EPAH-I was fractionated by DEAE-52 cellulose anion-exchange chromatography and separated into four fractions (EPAH-Ia to EPAH-Id) (Figure 3A). EPAH-Ia, EPAH-Ib, EPAH-Ic, and EPAH-Id were eluted out by deionized water, with 0.1, 0.25, and 0.5 M NaCl, respectively. At the concentration of 2.0 mg/mL, the scavenging activities of EPAH-Id on HO· and DPPH· were 56.38 ± 2.67% and 49.43 ± 0.94%, which were significantly higher than those of EPAH-I, EPAH-Ia, EPAH-Ib, and EPAH-Ic (Figure 3B). Anion-exchange chromatography is a method that separates compounds based on their charges using an ion-exchange resin containing positively charged groups [39,42]. Peptides with hydrophobic amino acid and/or basic residues, such as Leu, Ala, and Pro, are thought to have strong antioxidant activities and are usually isolated from protein hydrolysates using anion exchange resins [43,44]. Therefore, the present data indicated that EPAH-Id might contain high antioxidant amino acid residues and was chosen for further study.

**Figure 4:**
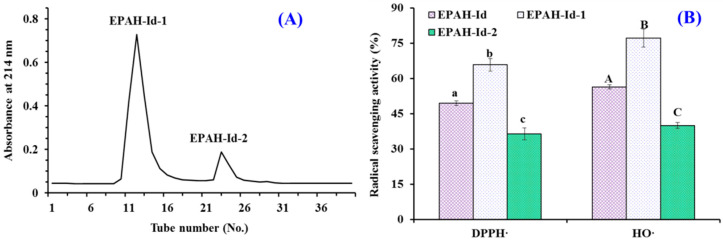
Sephadex G-25 chromatographic diagram (**A**) of EPAH-Id and the radical scavenging activity of EPAH-Id and its two fractions (EPAH-Id-1 and EPAH-Id-2) at 2.0 mg/mL (**B**). All the results were triplicates of mean ± SD. ^a–c or A–C^ Values with same superscripts of this type indicate no significant difference (*p* > 0.05).

Subsequently, EPAH-Id was separated by Sephadex G-25 gel chromatography on the basis of molecular size and two peptide fractions (EPAH-Id-1 and EPAH-Id-2) were prepared according to their chromatographic curves at 214 nm (Figure 4A). Figure 4B indicates that the HO· (77.17 ± 3.8%) and DPPH· (65.77 ± 0.83%) scavenging abilities of EPAH-Id-1 at the concentration of 2.0 mg/mL were significantly higher than those of EPAH-Id and EPAH-Id-2 (*p* < 0.05).

**Figure 5:**
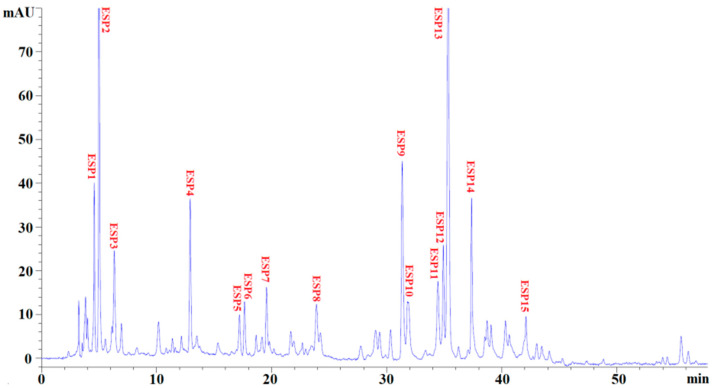
Elution profile of EPAH-Id-1 purified using RP-HPLC on a Zorbax SB C-18 column (4.6 mm × 250 mm) from 0 to 60 min at 214 nm.

Using the ultrafiltration and open column chromatography methods, the subfraction of EPAH-Id-1 with high HO· and DPPH· scavenging activity was finally separated using RP-HPLC with a linear gradient of acetonitrile and its peptide profifile at 214 nm, presented in Figure 5. Fifteen antioxidant peptides with retention times of 4.46 min (ESP1), 4.92 min (ESP2), 6.21 min (ESP3), 13.01 min (ESP4), 17.19 min (ESP5), 17.78 min (ESP6), 19.65 min (ESP7), 23.84 min (ESP8), 31.41 min (ESP9), 31.93 min (ESP10), 34.46 min (ESP11), 34.97 min (ESP12), 35.34 min (ESP13), 37.38 min (ESP14), and 42.17 min (ESP15), respectively, were isolated, collected and lyophilized (Table 1).

### 2.3. Identification of Antioxidant Peptides (ESP1 to ESP15) from EPAH-Id-1

Fifteen antioxidant peptides from EPAH-Id-1 (ESP1 to ESP15) underwent massive preparation through repeated RP-HPLC isolation, and their amino acid sequences and MWs were measured using a protein sequencer and ESI-MS. As shown in Table 1, their amino acid sequences were identified as Asn-Gln-Met (NQM, ESP1), Trp-Phe-Pro-Met (WFPM, ESP2), Gln-Asn- Pro-Thr (QNPT, ESP3), Tyr-Met-Asn-Phe (YMNF, ESP4), Ser-Gly-Pro-Ala (SGPA, ESP5), Ser-Leu-Pro-Tyr (SLPY, ESP6), Gln-Tyr-Pro-Pro-Met-Gln-Tyr (QYPPMQY, ESP7), Glu-Tyr-Glu-Ala (EYEA, ESP8), Asn-Trp-Asp-Asp-Met-Arg-Ile-Val-Ala-Val (NWDDMRIVAV, ESP9), Trp-Asp-Asp-Met-Glu-Arg-Leu-Val-Met-Ile (WDDMERLVMI, ESP10), Asn-Trp-Asp-Asp-Met-Glu-Pro-Ser-Phe (NWDDMEPSF, ESP11), Asn-Gly-Pro-Asp-Pro-Arg-Pro-Ser-Gln-Gln (NGPDPRPSQQ, ESP12), Ala-Phe-Leu-Trp-Asn (AFLWA, ESP13), Asn-Val-Pro-Asp-Met (NVPDM, ESP14), and Thr-Phe-Pro-Ile-Tyr-Asp-Tyr-Pro-Gln (TFPIYDPQ, ESP15), and their determined MWs using ESI-MS were well in line with their theoretical MWs (Table 1).

### 2.4. Antioxidant Activity of Antioxidant Peptides (ESP1 to ESP15)

For evaluating the antioxidant activities of isolated antioxidant peptides (ESP1 to ESP15), assays of radical scavenging, protective activity against radical-induced DNA damage, lipid peroxidation inhibition and reducing power were carried out, and GSH was employed as the positive control. The EC_50_ values of fifteen antioxidant peptides (ESP1 to ESP15) on HO·, DPPH·, and $O_{2}^{-}$· are shown in Table 2.

#### 2.4.1. Radical Scavenging Activity of Antioxidant Peptides (ESP1 to ESP15)

In the organism, HO· can oxidize and damage most of the macromolecules due to its high reactivity characteristic. Therefore, looking for natural antioxidant peptides with high HO· scavenging ability is key for discovering new antioxidants. Table 2 shows that EC_50_ values of ESP6, ESP7, and ESP8 on HO· were 0.826 ± 0.027, 1.022 ± 0.058, and 0.946 ± 0.011 mg/mL, respectively, which were significantly lower than those of the other twelve antioxidant peptides (*p* < 0.05), but there were significantly higher than that of GSH (*p* < 0.05). In addition, EC_50_ values of ESP6, ESP7, and ESP8 were also lower than those of antioxidant peptides from miiuy croaker swim bladders (2.31 ± 0.12, 2.35 ± 0.22, 2.45 ± 0.25, and 2.85 ± 0.19 mg/mL for FTGMD, GFYAA, FSGLR, and VPDD, respectively) [39], weatherfish loach (PSYV: 2.64 mg/mL) [45], skate cartilages (IVAGPQ: 5.03 mg/mL) [18], and heads of bluefin leatherjacket (WEGPK: 5.567 mg/mL) [42]. However, EC_50_ values of ESP6, ESP7, and ESP8 were also higher than those of antioxidant peptides from blue mussel (YPPAK: 0.228 mg/mL) [36], miiuy croaker swim bladders (FPYLRH: 0.68 ± 0.05 mg/mL) [39], *Sphyrna lewini* muscle (0.15 and 0.24 mg/mL for WDR and PYFNK, respectively) [46], and giant squid protein (0.123 and 0.078 mg/mL for NGPLQAGQPGER and FDSGPAGVL, respectively) [47,48]. The present finding indicated that the antioxidant peptides from Antarctic krill proteins, especially ESP6, ESP7, and ESP8, could efficiently scavenge HO· to decrease or clear off the damage induced by HO· in biological systems.

Table 2 indicates that ESP6 with EC_50_ value of 1.18 ± 0.036 mg/mL exhibited the strongest DPPH· scavenging ability among fifteen antioxidant peptides (ESP1 to ESP15), but the EC_50_ value of ESP6 was not significantly different to those of ESP1 (1.695 ± 0.033 mg/mL), ESP4 (1.672 ± 0.044 mg/mL), ESP7 (1.547 ± 0.150 mg/mL), and ESP8 (1.372 ± 0.274 mg/mL), but significantly lower than those of other ten isolated antioxidant peptides (*p* < 0.05). Moreover, the EC_50_ values of ESP1, ESP4, ESP6, ESP7, and ESP8 were less than those of antioxidant peptides from *Mytilus edulis* (YPPAK: 2.62 mg/mL) [36], weather loach (PSYV: 17.0 mg/mL) [45], *Sphyrna lewini* muscle (3.63 and 4.11 mg/mL for WDR and PYFNK, respectively) [46], and red stingray cartilages (4.01, 4.61, and 3.69 mg/mL for IEEEQ, VPR, LEEEE, respectively) [49]. However, the EC_50_ values of ESP1, ESP4, ESP6, ESP7, and ESP8 were higher than those of antioxidant peptides from monkfish muscle (0.39, 0.62, and 0.51 mg/mL for EDIVCW, MEPVW, and YWDAW, respectively) [22], scales of croceine croaker (0.675 and 0.283 mg/mL for GPAGPAG and GFPSG, respectively) [36], and miiuy croaker swim bladders (0.51 ± 0.03 and 0.78 ± 0.05 mg/mL for FPYLRH and GIEWA, respectively) [39]. These results indicated that the antioxidant peptides of ESP1, ESP4, ESP6, ESP7, and ESP8 had strong ability to inhibit DPPH· reaction.

Table 2 showed that the EC_50_ values of ESP6, ESP7, and ESP8 on $O_{2}^{-}$· were 0.789 ± 0.079, 0.913 ± 0.007, and 0.793 ± 0.056 mg/mL, respectively, which indicated that their $O_{2}^{-}$· scavenging ability was significantly stronger than those of other twelve antioxidant peptides (*p* < 0.05). However, no significant difference was found among the EC_50_ values of ESP6, ESP7, and ESP8 (*p* > 0.05). In addition, the EC_50_ values of ESP6, ESP7, and ESP8 were lower than those of antioxidant peptides from *Raja porosa* cartilage (1.61, 1.66, and 1.82 mg/mL for FIMGPY, GPAGDY, and IVAGPQ, respectively) [18], monkfish muscle (MEPVW: 0.94 mg/mL) [22], croceine croaker muscle (MILMR: 0.993 mg/mL) [37], swim bladders of miiuy croaker (3.04 ± 0.27, 3.61 ± 0.25, 3.03 ± 0.19, 3.35 ± 0.20, and 4.11 ± 0.31 mg/mL for FTGMD, YLPYA, GFYAA, FSGLR, and VPDDD, respectively) [39] and muscle (YFLWP: 3.08 mg/mL) [50]. However, The EC_50_ values of ESP6, ESP7, and ESP8 were higher than those of antioxidant peptides from blue mussel (YPPAK: 0.072 mg/mL) [36], *Sphyrna lewini* muscle (0.09 and 0.11 mg/mL for WDR and PYFNK, respectively) [46], and skipjack tuna heads (0.56, 0.38, and 0.71 mg/mL for WMFDW, WMGPY, and EMGPA, respectively) [25]. In organisms, $O_{2}^{-}$· can be transformed into the highly reactive HO∙ and toxic peroxy radicals, which will cause injury to some key biomolecules and further induce oxidative stress. Finally, oxidative stress causes the dysfunction of the organisms, and those influences have been strongly associated with the occurrence and evolution of some chronic diseases [17]. Therefore, ESP6, ESP7, and ESP8 might take on a major responsibility in clearing off $O_{2}^{-}$· damage in biological systems.

#### 2.4.2. Protective Activity of ESP6, ESP7, and ESP8 against H_2_O_2_-Damaged Plasmid DNA

**Figure 6:**
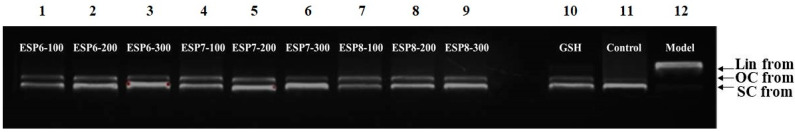
The protective effects of ESP6, ESP7, and ESP8 on the H_2_O_2_-damaged plasmid DNA (pBR322DNA). Lane 1, DNA + FeSO_4_ + H_2_O_2_ + ESP6 (100 µM); Lane 2, DNA + FeSO_4_ + H_2_O_2_ + ESP6 (200 µM); Lane 3, DNA + FeSO_4_ + H_2_O_2_ + ESP6 (300 µM); Lane 4, DNA + FeSO_4_ + H_2_O_2_ + ESP7 (100 µM); Lane 5, DNA + FeSO_4_ + H_2_O_2_ + ESP7 (200 µM); Lane 6, DNA + FeSO_4_ + H_2_O_2_ + ESP7 (300 µM); Lane 7, DNA + FeSO_4_ + H_2_O_2_ + ESP8 (100 µM); Lane 8, DNA + FeSO_4_ + H_2_O_2_ + ESP8 (200 µM); Lane 9, DNA + FeSO_4_ + H_2_O_2_ + ESP8 (300 µM); Lane 10, DNA + FeSO_4_ + H_2_O_2_ + GSH (200 µM); Lane 11, the native pBR322DNA; Lane 12, pBR322DNA + FeSO_4_ + H_2_O_2_.

DNA damage caused by superfluous ROS in organism is a key point in these ROS-induced degenerative processes, such as premature aging, neurodegenerative and cardiovascular diseases [24,51]. Then, the protective effects of ESP6, ESP7, and ESP8 against H_2_O_2_-damaged plasmid DNA (pBR322DNA) were measured and the results are shown in Figure 6. Under normal conditions, the supercoiled (SC) form is the main structure of plasmid DNA (pBR322 DNA) (Figure 6, lane 11). However, a relaxed open circular (OC) form will be generated when one of the phosphodiester chains of pBR322 DNA is split. Moreover, the linear (LIN) double-stranded DNA molecule is produced when second cleavage is near the first breakage. In the assay, HO· was generated from the chemical reaction of FeSO_4_ and H_2_O_2_, and it further cut off the DNA strands and converted the SC form into the OC form and LIN form structures [24,51,52]. Lane 12 in Figure 6 showed that the SC form of plasmid DNA (pBR322 DNA) was mainly converted into LIN form, which indicated that excessive HO· generated in the reaction broke the double strand of pBR322 DNA. As shown in Figure 6 (lane 1 to lane 9), the contents of the SC form of pBR322 DNA was obviously higher than that of the model group (Figure 6, lane 11). In addition, the contents of the SC form of pBR322 DNA in ESP6, ESP7, and ESP8 groups showed a significant concentration–effect relationship. In the high-dose groups, ESP6, ESP7, and ESP8 achieved similar protective effects to that of the positive control group (Figure 6, lane 10). These data indicated that ESP6, ESP7, and ESP8 have significant protective ability on plasmid DNA (pBR322 DNA). These results were also in line with the finding in Table 2 that ESP6, ESP7, and ESP8 could effectively scavenge HO· to protect biomolecules.

#### 2.4.3. Lipid Peroxidation Inhibition Assay of ESP6, ESP7, and ESP8

**Figure 7:**
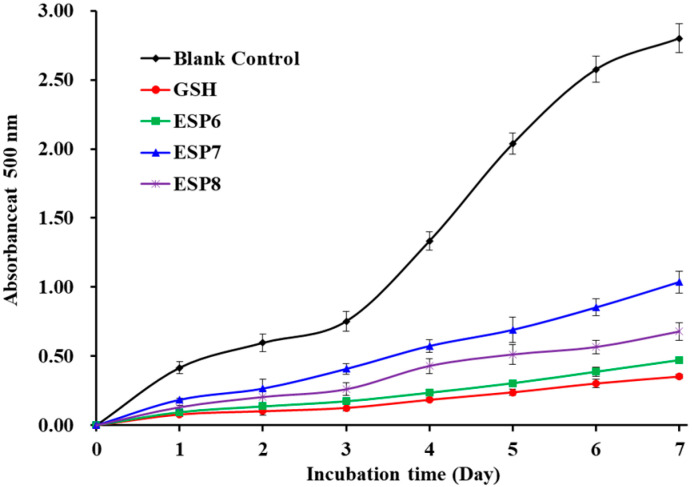
Lipid peroxidation inhibition capability of ESP6, ESP7, and ESP8 from the alcalase hydrolysate of Antarctic krill (*E. superba*) proteins. All the results were triplicates of mean ± SD.

Lipid peroxidation is a key factor of the aging process and pathophysiology of many chronic diseases [42]. In addition, lipid peroxidation can change food properties, including nutrition, texture, flavor, color, and luster, to gives rise to food deterioration. In the linoleic acid system, the lipid peroxidation inhibition abilities of ESP6, ESP7, and ESP8 were measured and the lower absorbance at 500 nm illustrates higher antioxidant ability [25]. Figure 7 indicates the absorbance value of the blank control group was significantly higher than those of antioxidant peptides (ESP6, ESP7, and ESP8) and the positive control (GSH) groups, which indicated that ESP6, ESP7, ESP8, and GSH can effectively control the peroxidation reaction when they were incubated with linoleic acid for 7 days. Furthermore, the absorbance values of the ESP6 group were lower than those of ESP7 and ESP8 groups. Then, ESP6, ESP7, and ESP8 could serve as the natural antioxidant agents to control lipid oxidation in organisms and fat-rich foods.

#### 2.4.4. Reducing Power of ESP6, ESP7, and ESP8

**Figure 8:**
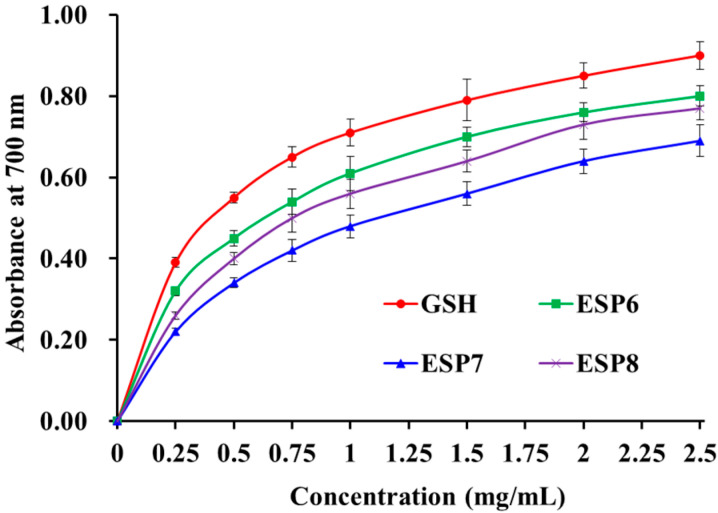
The reducing power of ESP6, ESP7, and ESP8 from the alcalase hydrolysate of Antarctic krill (*E. superba*) proteins. All the results were triplicates of mean ± SD.

As presented in Figure 8, the reducing power of ESP6, ESP7, and ESP8 showed a certain dose–effect relationship when their concentrations were decreased from 0 to 2.5 mg/mL. The result illustrated that ESP6 had a higher reducing capacity to convert Fe^3+^/ferricyanide complex into the ferrous form than ESP7 and ESP8. However, the reducing power of ESP6, ESP7, and ESP8 was less than that of GSH. The reducing power is considered to be an important indicator of the potential activity of antioxidant peptides in reduction reactions, which can be used to evaluate their abilities to provide hydrogen and/or electrons [41,50]. The present results suggested that ESP6, ESP7, and ESP8 could serve as electron donors to reduce the oxidized intermediates in lipid peroxidation reactions of organisms.

Antioxidant peptides have been prepared from different food protein resources. Molecular size is believed to play an essential role in their activities [11]. Antioxidant peptides with smaller molecules can be more accessible to free radicals to control the cycle of lipid peroxidation [11,53]. Moreover, molecular size is an important constraint for antioxidant peptides striding over the blood–brain barrier to exert their pharmacological activities in living organisms [54]. Therefore, ESP6, ESP7, and ESP8 could be easily close to the free radical to play their functions, because they were oligoptides with MWs of 478.80, 926.00, and 510.90 Da, respectively (Table 1).

Amino acid composition, including species, sequence and spatial structure, is another key factor thought to be involved in the bioactivities of antioxidant peptides [11]. Hydrophobic and aromatic amino acids, such as Leu, Pro, Tyr, Phe, Ala, and Met, have an important impact on the activity of antioxidant peptides because those amino acids could raise the antioxidant activities through improving the lipid solubility and combination with radical species of antioxidant peptides [11,21]. Rajapakse et al. (2005) and Gulçin (2007) reported that Leu showed the highest antioxidant activity among the amino acids [55,56]. Chen et al. (2020) speculated that Leu and Thr contribute to the highest activity of ILGATIDNSK from defatted round scad [57]. Yang et al. (2019) reported that Ala, Ile, and Val in GADIVA, and Ala and Ile in GAEGFIF could assist them in a conducive manner to combine with target radicals [26]. Wu et al. (2018) reported that Met residue contributed greatly to the inhibition of free-radical chain reactions of PMRGGGGYHY because it could format a sulfoxide structure, which acted as a reactive site to clear oxidants [58]. Therefore, Leu, Met, and Ala should play a key role in the antioxidant activity of ESP6, ESP7, and ESP8, respectively.

Wong et al. (2020) reported that Pro could serve as a proton/hydrogen donor to play its antioxidant role [59]. In addition, Pro could increase the flexibility of bioactive peptides, and the low ionization potential of its pyrrolidine ring could quench singlet oxygen [53]. Therefore, Pro presented in ESP6 and ESP7 are important for their bioactivities. Tyr residues could remove free radicals and provide protons to electron-deficient radicals to change them into more stable phenoxy radicals, which could inhibit the peroxidizing serial reaction induced by ROS during the scavenging process [60,61]. Wu et al. (2018) and Sheih et al. (2009) reported that Tyr could act as hydrogen donors to play an important role in antioxidant activity, because it could remove free radicals and change them into more stable phenoxy radicals, which inhibited the propagation of the radical-mediated peroxidizing chain reaction [58,61]. Guo et al. (2009) reported that antioxidant peptides with Tyr residues (YDY, RY, YEEN, KY, YEG, YD, YY, and RYN) showed high antioxidant ability [62]. Then, Tyr is contributed to the bioactivity of ESP6, ESP7, and ESP8.

In the lipid and aqueous solutions of oxidation, the ratio of hydrophilic/hydrophobic amino acids in antioxidant peptides can significantly affect their solubility and biological activity [21,63]. Therefore, the hydrophobic amino acids were critical for their protective response in lipid peroxidation by eliminating the free radicals derived from lipids in a heterogeneous lipid phase [64]. Gly residue can maintain the strong flexibility of the polypeptide skeleton and act as single hydrogen donor to neutralize ROS [25,65]. Hydrophilic amino acids including Glu, Gln, and Lys in EVGK and RCLQ have a positive influence on their Fe^2+^ chelating ability [65]. Asp, Glu, and Gln were found to show remarkable influence on the antioxidant abilities of NYDGSTDYGILQINSR and LDEPDPLI [66,67]. Therefore, Ser, Gln, and Glu are also contribute to the radical-scavenging, lipid peroxidation inhibitory, and reducing power ability of ESP6, SP7, and ESP8, respectively.

### 2.5. Effects of pH, Thermal, and Simulated GI Digestion Treatments on the Stability of ESP6, ESP7, and ESP8

**Figure 9:**
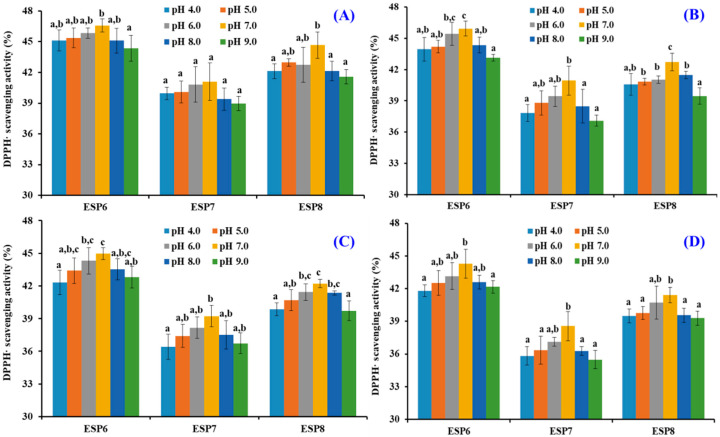
DPPH· scavenging activity of ESP6, ESP7, and ESP8 subjected to different pH treatments for 30 min (**A**), 60 min (**B**), 120 min (**C**), and 180 min (**D**), respectively. All the results were triplicates of mean ± SD. ^a–c^ values with same letters indicate no significant difference of same peptide subjected to different pH treatments (*p* > 0.05).

Figure 9 indicates that the DPPH· scavenging activity of ESP6, ESP7, and ESP8 treated with designated pH values has the same varying tendency. Under neutral conditions, ESP6, ESP7, and ESP8 showed the highest activity. In addition, it could be found that the DPPH· scavenging activity of ESP6, ESP7, and ESP8 decreased gradually with the time from 0 to 180 min. Except 30 min, DPPH· scavenging activity of ESP6, ESP7, and ESP8 subjected to pH 7.0 treatment was significantly higher than those of ESP6, ESP7, and ESP8 subjected to pH 4.0 and 9.0 treatments. The results indicated that high acid and alkali treatments had significant negative effects on the antioxidant activity of ESP6, ESP7, and ESP8. Table 3 shows that the reduced proportion of ESP6 treated for 180 min at different pH values was smaller than those of ESP7 and ESP8, which indicated that the pH stability of ESP6 was higher than those of ESP7 and ESP8.

**Figure 10:**
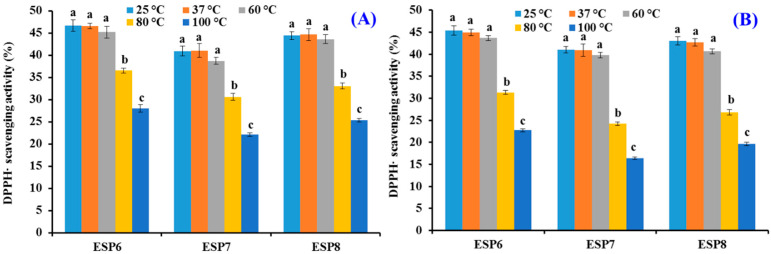
DPPH· scavenging activity of ESP6, ESP7, and ESP8 subjected to different thermal treatments for 30 min (**A**) and 60 min (**B**). All the results were triplicates of mean ± SD. ^a–c^ Values with same letters indicate no significant difference of same peptide subjected to different temperature treatments (*p* > 0.05).

As shown in Figure 10, different temperature treatments could greatly affect the DPPH· scavenging activities of ESP6, ESP7, and ESP8. The DPPH· scavenging activities of ESP6, ESP7, and ESP8 at 25, 37, and 60 °C for 30 and 60 min were significantly different from those treated at 80 and 100 °C for 30 and 60 min, respectively (*p* < 0.05). In addition, there was no significant difference of the DPPH· scavenging activities of ESP6, ESP7, and ESP8 at 25, 37, and 60 °C for 30 and 60 min (*p* > 0.05). In addition, the DPPH· scavenging activities of ESP6, ESP7, and ESP8 decreased by 51.42 ± 2.76%, 60.26 ± 3.51%, and 56.01 ± 1.98%, respectively, at 100 °C when the time was ranged from 0 min to 60 min. Those data suggested that ESP6 was more tolerant to high temperatures compared with ESP7 and ESP8.

**Figure 11:**
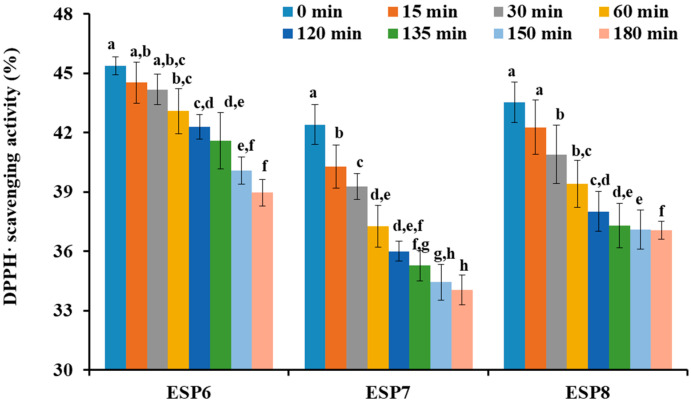
DPPH· scavenging activity of ESP6, ESP7, and ESP8 subjected to simulated GI digestion treatments from 0 to 180 min. All the results were triplicates of mean ± SD. ^a–h^ Values with same letters indicate no significant difference of same sample at different time (*p* > 0.05).

In response to simulated GI digestion, the DPPH· scavenging ratios of ESP6, ESP7, and ESP8 are shown in Figure 11. The data indicated that DPPH· scavenging activities of ESP6, ESP7, and ESP8 at the concentration of 1.0 mg/mL were decreased gradually with the treating time ranged from 0 to 180 min. The DPPH· scavenging activities of ESP6, ESP7, and ESP8 before simulated GI digestion (ESP6: 45.39 ± 0.46%; ESP7: 42.41 ± 1.01%; ESP8: 43.54 ± 1.02%) were significantly (*p* < 0.05) lower than those obtained after simulated GI digestion (ESP6: 38.97 ± 0.67%; ESP7: 34.05 ± 0.74%; ESP8: 37.06 ± 0.45%). Furthermore, the DPPH· scavenging ratios of ESP6, ESP7, and ESP8 decreased by 6.41 ± 0.96%, 8.36 ± 0.59%, and 6.48 ± 1.03%, respectively, which suggested that ESP6 has stronger stability than ESP7 and ESP8 when they were treated with simulated GI diges tion.

The pH and thermal stability of antioxidant peptides are key properties for their applications in food and health products, and tolerance property on simulated GI digestion treatments can help to assess the rate of metabolism and timeliness of antioxidant peptides in vivo [25,52,68,69]. The previous literature indicated that WAFAPA and MYPGLA with 25–100 °C or pH 3–11 treatments show high stability [59], but ATSHH treated with 50–90 °C and strong acids and bases will decrease its partial DPPH· scavenging activity [70]. Zhang et al. (2019) found that the HO· scavenging activity of WMFDW, WMGPY, and EMGPA could be significantly influenced under high temperature (>60 °C) and strong acid and alkali conditions [25]. The present results indicated that ESP6 (SLPY), ESP7 (QYPPMQY), and ESP8 (EYEA) had similar thermal and pH stability to ATSHH, WMFDW, WMGPY, and EMGPA because their activity was significantly decreased under high temperature (>60 °C) and strong acid and alkali conditions. In addition, the stability of ESP6 (SLPY) was higher than those of ESP7 (QYPPMQY) and ESP8 (EYEA) under thermal, pH, and simulated GI digestion treatments.

## 3. Materials and Methods

### 3.1. Materials

Antarctic krill (*E. superba*) powder was kindly provided by Zhejiang Hailisheng Biotechnology Co. Ltd. (Zhoushan, China). Trypsin, papain, trifluoroacetic acid, 2,2-Diphenyl-1-picrylhydrazyl (DPPH), phosphate buffered saline, Sephadex G-25, and pepsin, were purchased from Sigma-Aldrich (Shanghai) Trading Co., Ltd. (Shanghai, China). Neutrase was purchased from Imperial Jade Biotechnology, Co. Ltd. (Yinchuan, China). Alcalase was purchased from Novozymes Biotechnology Co., Ltd. (Tianjin, China). Diethylaminoethyl (DEAE)-52 cellulose anion exchange resin was purchased from Nanjing Jiancheng Bioengineering Co., Ltd. (Nanjing, China). Acetonitrile was bought from Thermo Fisher Scientific (Shanghai) Co., Ltd. (Shanghai, China).

### 3.2. Preparation of Protein Hydrolysate of Antarctic Krill (EPAH)

The Antarctic krill powder was defatted according to our previous method with a light modification [4]. Subsequently, the defatted shrimp powder was dissolved in 0.05 M phosphate buffer with a solid/liquid ratio of 1:30, and the mixed solutions were treated for 6 h separately using pepsin (pH 2.0, 37 °C), alcalase (pH 8.5, 50 °C), papain (pH 6.0, 50 °C), neutrase (pH 7.0, 60 °C), and trypsin (pH 8.0, 40 °C) with total dosage of enzyme of 2.0% (*w*/*w*). After hydrolysis reaction, five hydrolysates were kept in a boiling water bath for 15 min to inactivate enzymes and centrifuged at 4000× *g* for 15 min. The supernatants of five hydrolysates were concentrated, lyophilized and kept in a −20 °C refrigerator. In addition, alcalase hydrolysate was named EPAH.

### 3.3. Preparation of Antioxidant Peptides from Alcalase Hydrolysate (EPAH) of Antarctic Krill Proteins

**Figure 12:**
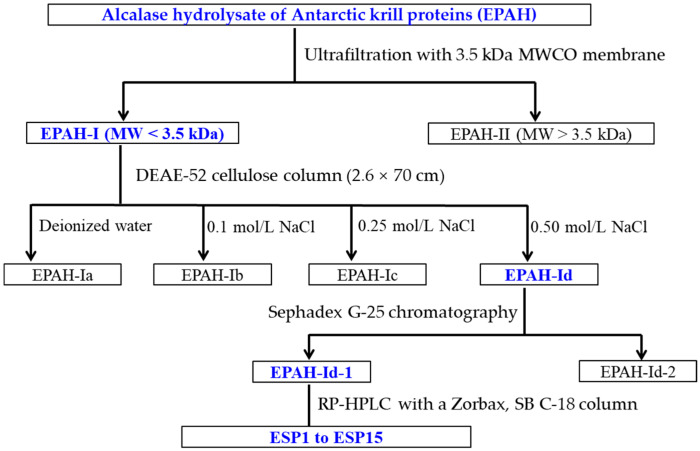
The flow chart of purifying antioxidant peptides from alcalase hydrolysate (EPAH) of Antarctic krill (*E. superba*) proteins.

Antioxidant peptides were purified from EPAH by the ultrafiltration and chromatographic process (Figure 12).

EPAH was ultrafiltrated by 3.5 kDa molecular weight (MW) cut-off membrane, and two peptide fractions of EPAH-I (MW < 3.5 kDa) and EPAH-II (MW > 3.5 kDa) were prepared.

EPAH-I solution (6 mL, 50.0 mg/mL) was loaded onto a DEAE-52 cellulose column (2.6 × 70 cm) pre-equilibrated with deionized water, and eluted with deionized water, and a 0.10, 0.25, and 0.50 M NaCl solution, respectively. The flow rate of eluent was set as 3.0 mL/min and monitored at 214 nm. Four fractions (EPAH-Ia to EPAH-Id) were collected according to chromatogram map.

An amount of 5 mL of EPAH-Id solution (50.0 mg/mL) was injected into the Sephadex G-25 chromatographic column (2.6 cm × 120 cm) and eluted using ultrapure water. The eluent with a flow rate of 0.6 mL/min was collected every 3 min and two components, named EPAH-Id-1 and EPAH-Id-2, were isolated according to the chromatographic curve of EPAH-Id at 214 nm.

EPAH-Id-1 was further separated by the Agilent 1200 HPLC system (Agilent Ltd., Santa Clara, CA, USA) on a Zorbax, SB C-18 column (4.6 × 250 mm, 5 µm) using a linear gradient of acetonitrile (0–100% in 0–60 min) in 0.05% trifluoroacetic acid. The elution solution with a flow rate of 0.8 mL/min was monitored at 214 nm. Finally, fifteen antioxidant peptides (ESP1 to ESP15) were isolated from EPAH-Id-1 on their chromatographic peaks, lyophilized, and kept in a −20 °C refrigerator.

### 3.4. Identification of Antioxidant Peptides (ESP1 to ESP15) from EPAH-Id-1

The Applied Biosystems 494 protein sequencer (Perkin Elmer/Applied Biosystems Inc., Foster City, CA, USA) was employed to analyze the N-terminal amino acid sequences of fifteen antioxidant peptides (ESP1 to ESP15), and the Q-TOF mass spectrometer (MS) (Micromass, Waters, Milford, MA, USA) with an electrospray ionization (ESI) source were applied to measure the MWs of fifteen antioxidant peptides (ESP1 to ESP15).

### 3.5. Antioxidant Activities of Antioxidant Peptides (ESP1 to ESP15)

The scavenging assays of DPPH radical (DPPH·), superoxide anion radical ($O_{2}^{-}$·), and hydroxyl radical (HO·) were determined on the previous method [35,36], and the EC_50_ values of antioxidant peptides (ESP1 to ESP15) on DPPH·, HO·, and $O_{2}^{-}$· were defined as the sample concentration induced half of the decrease in the initial radical contents. The assays of reducing power and lipid peroxidation inhibition were performed according to the methods described by Zhang et al. (2019) [25], and GSH was used as the positive control in all antioxidant assays.

### 3.6. Stability Characteristics of ESP6, ESP7, and ESP8 against the Treatments of Heat, pH, and Simulated Gastrointestinal (GI) Digestion

The pH stability and thermostability of ESP6, ESP7, and ESP8 were analyzed in accordance with the previous method with a light modification [25]. In short, effects of acid and alkali treatments at pH values of 5, 6, 7, 8, or 9 were employed to estimate the acid and alkali stability characteristics of ESP6, ESP7, and ESP8 at 25 °C, and the analyzed time was set to 30, 60, 120, and 180 min.

The thermostability of ESP6, ESP7, and ESP8 at 25, 37, 60, 80, or 100 °C was analyzed in water bath, and the analyzed time was set to 30 and 60 min.

The influence of simulate GI digestion on the stability of ESP6, ESP7, and ESP8 was evaluated by the two-stage digestion model [26]. In short, ESP6, ESP7, and ESP8 were separately treated with pepsin for 120 min and pancreatin for 60 min.

The DPPH· scavenging ratio (%) of ESP6, ESP7, and ESP8 at 1.0 mg/mL were measured at the set time to analyze their stability.

### 3.7. Statistical Analysis

The experiment data were represented as the mean ± standard deviation (SD, *n* = 3). An ANOVA test using SPSS 19.0 (SPSS Corporation, Chicago, IL, USA) was employed to analyze the means of each treatment, and Duncan’s multiple range test was applied to analyze the significant differences among the different groups (*p* < 0.05).

## 4. Conclusions

In summary, the purification, identification, activity evaluation and stability of antioxidant peptides from alcalase hydrolysate of Antarctic krill (*E. superba*) proteins were systematically studied, and fifteen antioxidant peptides were purified from alcalase hydrolysate and identified as NQM, WFPM, QNPT, YMNF, SGPA, SLPY, QYPPMQY, EYEA, NWDDMRIVAV, WDDMERLVMI, NWDDMEPSF, NGPDPRPSQQ, AFLWA, NVPDM, and TFPIYDPQ, respectively. Among them, SLPY, QYPPMQY and EYEA showed high radical scavenging activities, H_2_O_2_-damaged plasmid DNA protective effects, reducing power, and lipid peroxidation inhibition ability. In addition, SLPY, QYPPMQY, and EYEA had high stability under temperatures lower than 80 °C, pH values ranged from 6–8, and simulated GI digestion for 180 min. The present results provided support for SLPY, QYPPMQY and EYEA to serve as effective antioxidant agents used in health-promoting food products. The antioxidant mechanism in vivo models and the clinical efficacy of SLPY, QYPPMQY and EYEA have been researched in our lab.

## Figures and Tables

**Table 1:** Retention time, amino acid sequences, and MWs of fifteen antioxidant peptides (ESP1 to ESP15) from alcalase hydrolysate of Antarctic krill (*E. superba*) proteins.

	Retention Time (min)	Amino Acid Sequence	Theoretical MW/Determined MW (Da)
ESP1	4.46	NQM	391.44/391.50
ESP2	4.92	WFPM	579.71/580.03
ESP3	6.21	QNPT	458.47/458.50
ESP4	13.01	YMNF	573.66/573.50
ESP5	17.19	SGPA	330.34/330.15
ESP6	17.78	SLPY	478.54/478.80
ESP7	19.65	QYPPMQY	926.05/926.00
ESP8	23.84	EYEA	510.49/510.60
ESP9	31.41	NWDDMRIVAV	1218.38/1218.40
ESP10	31.93	WDDMERLVMI	1307.54/1307.40
ESP11	34.46	NWDDMEPSF	1140.18/1140.30
ESP12	34.97	NGPDPRPSQQ	1094.14/1094.21
ESP13	35.34	AFLWA	649.74/650.10
ESP14	37.38	NVPDM	574.65/574.60
ESP15	42.17	TFPIYDPQ	1143.24/1143.30

**Table 2:** EC_50_ values of fifteen antioxidant peptides (ESP1 to ESP15) from alcalase hydrolysate of Antarctic krill (*E. superba*) proteins on HO·, DPPH·, and O− 2·, respectively.

	Amino Acid Sequence	EC_50_ (mg/mL)		
		HO·	DPPH·	$O_{2}^{-}$·
ESP1	NQM	1.425 ± 0.067 ^a^	1.695 ± 0.033 ^a,e,i^	1.796 ± 0.029 ^a^
ESP2	WFPM	1.751 ± 0.075 ^b^	5.364 ± 0.337 ^b^	2.746 ± 0.302 ^b^
ESP3	QNPT	1.931 ± 0.031 ^c,h^	7.193 ± 0.460 ^c^	2.933 ± 0.075 ^c^
ESP4	YMNF	1.443 ± 0.066 ^a,j^	1.672 ± 0.044 ^a,e,i^	1.136 ± 0.063 ^d^
ESP5	SGPA	2.362 ± 0.021 ^d^	4.135 ± 0.192 ^d^	1.863 ± 0.104 ^a,e^
ESP6	SLPY	0.826 ± 0.027 ^e^	1.181 ± 0.036 ^e^	0.789 ± 0.079 ^f^
ESP7	QYPPMQY	1.022 ± 0.058 ^f^	1.547 ± 0.150 ^a,e^	0.913 ± 0.007 ^f^
ESP8	EYEA	0.946 ± 0.011 ^e,f^	1.372 ± 0.274 ^a,e^	0.793 ± 0.056 ^f^
ESP9	NWDDMRIVAV	2.612 ± 0.013 ^g^	6.192 ± 0.192 ^f^	3.756 ± 0.025 ^g^
ESP10	WDDMERLVMI	1.953 ± 0.042 ^h^	4.719 ± 0.163 ^g^	1.996 ± 0.011 ^e^
ESP11	NWDDMEPSF	2.598 ± 0.036 ^g^	3.029 ± 0.077 ^h^	1.862 ± 0.094 ^a,e^
ESP12	NGPDPRPSQQ	2.742 ± 0.105 ^i^	7.054 ± 0.460 ^c^	2.031 ± 0.011 ^e^
ESP13	AFLWA	1.527 ± 0.080 ^j,k^	2.029 ± 0.092 ^i^	1.162 ± 0.036 ^d^
ESP14	NVPDM	1.839 ± 0.032 ^b,c^	4.876 ± 0.145 ^g^	2.916 ± 0.153 ^c^
ESP15	TFPIYDPQ	1.549 ± 0.072 ^k^	2.135 ± 0.106 ^i^	1.252 ± 0.051 ^d^
Positive control	GSH	0.492 ± 0.063 ^l^	0.073 ± 0.021 ^j^	0.250 ± 0.023 ^h^

All the results were triplicates of mean ± SD. ^a–^^l^ Values with same superscripts indicate no significant difference of different peptide on same radicals (*p* > 0.05).

**Table 3:** Reduced proportion of DPPH· scavenging activity of ESP6, ESP6, and ESP8 subjected to different pH treatments for 180 min.

	Reduced Proportion of DPPH· Scavenging Activity (%)					
	pH 4.0	pH 5.0	pH 6.0	pH 7.0	pH 8.0	pH 9.0
**ESP6**	5.76 ± 0.06	5.06 ± 0.55	4.42 ± 0.73	3.29 ± 0.77	4.99 ± 0.58	5.41 ± 0.95
**ESP7**	6.95 ± 0.65	6.44 ± 0.79	5.65 ± 1.33	4.22 ± 1.34	6.49 ± 1.21	7.29 ± 0.99
**ESP8**	6.99 ± 6.72	6.72 ± 0.87	5.74 ± 1.36	5.05 ± 0.89	6.92 ± 0.99	7.19 ± 0.52

Data are expressed as mean ± SD (*n* = 3).

## Data Availability

Data are contained within the article.

## Abbreviations

ROS, reactive oxide species; EDIVCW, Glu-Asp-Ile-Val-Cys-Trp; YWDAW, Tyr-Trp-Asp-Ala-Trp; GSH, glutathione; FPYLRH, Phe-Pro-Tyr-Leu-Arg-His; HUVECs, human umbilical vein endothelial cells; MDA, malondialdehyde; ICRD, Ile-Cys-Arg-Asp; LCGEC, Leu-Cys-Gly-Glu-Cys; Keap1, Kelch-like ECH-associating protein 1; Nrf2, nuclear factor erythroid 2-related factor 2; ARE, antioxidant response element; EDYGA, Glu-Asp-Tyr-Gly-Ala; DPP-IV, dipeptidyl peptidase IV; ACE, angiotensin converting enzyme; FAS, Phe-Ala-Ser; KVEPLP, Lys-Val-Glu-Pro-Lys-Pro; PAL, Pro-Ala-Lys; IPA, Ile-Pro-Ala; VLGYIQIR, Val-Lys-Gly-Tyr-Ile-Gln-Ile-Arg; DEAE, diethylaminoethyl; DPPH, 2,2-Diphenyl-1-picrylhydrazyl; EPAH, the alcalase hydrolysate of *Euphausia superba* proteins; MW, molecular weight; ESI, electrospray ionization; MS, mass spectrometer; DPPH·, DPPH radical; HO·, hydroxyl radical; $O_{2}^{-}$, superoxide anion radical; GI, Gastrointestinal; FTGMD, Phe-Thr-Gly-Met-Asp; GFYAA, Gly-Phe-Tyr-Ala-Ala; FSGLR, Phe-Ser-Gly-Leu-Arg; VPDD, Val-Pro-Asp-Asp; PSYV, Pro-Ser-Tyr-Val; IVAGPQ, Ile-Val-Ala-Gly-Pro-Gln; WEGPK, Trp-Glu-Gly-Pro-Lys; YPPAK, Tyr-Pro-Pro-Ala-Lys; WDR, Trp-Asp-Arg; NGPLQAGQPGER, Asn-Gly-Pro-Leu-Gln-Ala-Gly-Gln-Pro-Gly-Glu-Arg; PYFNK, Pro-Tyr-Phe-Asn-Lys; FDSGPAGVL, Phe-Asp-Ser-Gly-Pro-Ala-Gly-Val-Leu; IEEEQ, Ile-Glu-Glu-Glu-Gln; VPR, Val-Pro-Arg; LEEEE, Leu-Glu-Glu-Glu-Glu; MEPVW, Met-Glu-Pro-Val-Trp; GPAGPAG, Gly-Pro-Ala-Gly-Pro-Ala-Gly; GFPSG, Gly-Phe-Pro-Ser-Gly; FPYLRH, Phe-Pro-Tyr-Leu-Arg-His; GIEWA, Gly-Ile-Glu-Trp-Ala; FIMGPY, Phe-Ile-Met-Gly-Pro-Tyr; GPAGDY, Gly-Pro-Ala-Gly-Asp-Tyr; MILMR, Met-Ile-Leu-Met-Arg; YLPYA, Tyr-Leu-Pro-Tyr-Ala; YFLWP, Tyr-Phe-Leu-Trp-Pro; WMFDW, Trp-Met-Phe-Asp-Trp; WMGPY, Trp-Met-Gly-Pro-Tyr; EMGPA, Glu-Met-Gly-Pro-Ala; ILGATIDNSK, Ile-Leu-Gly-Ala-Thr-Ile-Asp-Asn-Ser-Lys; GADIVA, Gly-Ala-Asp-Ile-Val-Ala; GAEGFIF, Gly-Sla-Glu-Gly-Phe-Ile-Phe; PMRGGGGYHY, Pro-Met-Arg-Gly-Gly-Gly-Gly-Tyr-His-Tyr; YDY, Tyr-Asp-Tyr; RY, Arg-Tyr; YEEN, Tyr-Glu-Glu-Asn; KY, Lys-Tyr; YEG, Tyr-Glu-Gly; YD, Tyr-Asp.
